# Spiritual needs and influencing factors among people with stroke in China: a cross-sectional study

**DOI:** 10.1186/s12912-024-02182-7

**Published:** 2024-07-29

**Authors:** Z.-Y. Li, X. Cao, S. Li, T.-J. Huang, Y.-X. Liu, L.-H. Qin

**Affiliations:** 1 School of Nursing, Hunan University of Chinese Medicine, Changsha, China; 2 Teaching and Research Section of Clinical Nursing, Xiangya Hospital Central South University, Changsha, China; 3 School of Informatics, Hunan University of Chinese Medicine, Changsha, China; 4 Key Laboratory of Hunan Province for Prevention and Treatment of Integrated Traditional Chinese and Western Medicine on Cardiocerebral Diseases, Hunan University of Chinese Medicine, Changsha, China

**Keywords:** Spiritual needs, Stroke, Quality of life, Anxiety, Depression, Family support

## Abstract

**Background:**

Stroke is one of the primary causes of disability in China and around the world, having an impact on the health and well-being of stroke patients. The importance of spiritual needs for stroke patients has always been a controversial topic internationally, partly because related research was mostly qualitative and may not directly reflect the degree of spiritual needs. In addition, most studies focus on the same cultural background, there is a lack of research that delves into the nuances of Chinese culture and background. The goal of this study is to evaluate the level of spiritual needs and influencing factors in Chinese stroke patients and to explore the mediating role and pathways of these variables on spiritual needs.

**Methods:**

From August 2022 to January 2023, we conducted a cross-sectional questionnaire survey of 422 stroke patients in the affiliated hospitals of Hunan University of Chinese Medicine in Changsha Province by cluster sampling. We measured the patient’s spiritual needs, quality of life, anxiety and depression levels, and family support using the Spiritual Needs Questionnaire (SPNQ), the MOS36 item Short Form Health Survey (SF-36), the Hospital Anxiety and Depression Scale (HADS), and the Family Support Self Rating Scale (PSS-Fa). We used the General Information Questionnaire to gain insight into the sociodemographic characteristics of the patients. Nonparametric tests and multiple linear regression models were used to analyze the independent relationship between spiritual needs and quality of life, anxiety, depression, and family support. The mediation model in AMOS 24.0 software was used to analyze the mediating role among the five variables.

**Results:**

The score of spiritual needs of people with stroke included in this study was 37 points [IQR 33 to 40)]. The influencing factors of spiritual needs included primary economic sources for disease-related expenditures (*p* = 0.044), number of stroke occurrences (*p* = 0.001), duration of illness (*p* = 0.023), activities of daily living (*p* = 0.006), depression scores(*p* = 0.034), and family support scores(*p* = 0.008). Anxiety (β = 0.347, *p* = 0.004), depression (β = 0.368, *p* = 0.005), and family support (β = 0.167, *p* = 0.023) had directly or indirectly affected the spiritual needs of people with stroke. Quality of life (β=-0.202, *p* = 0.017) had a direct effect on spiritual needs.

**Conclusions:**

The spiritual needs of people with stroke were at an intermediate level. Our findings highlight that the SPNQ score is associated with anxiety, depression, quality of life, and family support. Therefore, nurses should identify the spiritual needs of patients and provide them with effective and comprehensive spiritual care by reducing negative emotions and enhancing social support, promoting the development and progress of spiritual care in China. This study offers a theoretical basis for the spiritual care of clinical people with stroke and constructing a stroke spiritual care model.

**Supplementary Information:**

The online version contains supplementary material available at 10.1186/s12912-024-02182-7.

## Background

Stroke is an acute cerebrovascular disease characterized by sudden onset and rapid appearance of focal or global neurological deficits as the primary clinical manifestations. It has become a significant global health issue due to its high incidence, high mortality, and high disability rate [1]. The World Stroke Organization (WSO) pointed out that stroke affects approximately 11.4 million people worldwide every year, with 6.5 million deaths, making it the second leading cause of death globally [2, 3]. According to the Global Burden of Disease Study (GBD) in 2021, the incidence and prevalence of stroke have significantly increased in the population under the age of 70 (22% increase in incidence and 15% increase in prevalence), especially among women and the elderly. Population aging and unhealthy lifestyles are the main reasons for the increase in incidence and prevalence [4, 5]. Post-people with stroke often have functional impairments, including motor disorders such as hemiplegia, central facial palsy, and language and speech disorders [6]. Additionally, post-stroke anxiety and depression are common symptoms among survivors, with over one-third of patients experiencing post-stroke worry and anxiety [7]. Due to the recurrent nature of the disease and long-term prognosis, the sequelae of stroke seriously affect the physiological, psychological, and social well-being and quality of life of patients, and even lead to negative impacts on their perceived self-worth, which hurts patients’ health outcomes [8]. During this period, patients often urgently desire to seek their meaning, and goals, and get life value and expectation [9].

Spiritual needs refer to the demands and expectations of individuals in their search for meaning, goals, and values [10]. Research has indicated a direct correlation between patients’ spiritual needs and their ability to heal, and meeting these needs helps patients maintain good mental and physical health and positive self-management skills [11]. In the holistic care approach, spiritual needs are crucial [12]. The patient’s physical and mental health, social support, and other factors, such as their level of anxiety, depression, and family support, are all directly linked to their spiritual needs. It is beneficial for patients’ spiritual well-being and physical and mental health to meet their spiritual needs. Higher spirituality has been linked to better outcomes for people with stroke, including the promotion of healthy behaviors and a reduction in depressive symptoms, according to prior research [13]. Although it had been suggested that people with stroke have unmet spiritual needs [14], the majority of earlier research on the subject was qualitative. With the increasing attention of the academic community to spiritual needs, several scholars had conducted empirical research on the spiritual needs of stroke patients. Oshvandi conducted an intervention study on spiritual care programs for stroke patients in Iran and found that spiritual care increased the hope of stroke patients, emphasizing that nurses should recognize the spiritual needs of stroke patients [15], another research conducted by Yousofvand indicated that effective spiritual care programs could improve the spiritual health of stroke patients [16]. However, most of these studies were limited to the same region or cultural context. There is a lack of research based on the nuances of Chinese culture and context, and identifying and addressing this research gap is critical to developing holistic care in different cultures and Settings. Thus, additional empirical research in various cultural contexts is required to support the spiritual needs of people with stroke as well as associated risk factors.

Therefore, we investigated the spiritual needs and the factors influencing people with stroke in China during illness and recovery. It aims to analyze the factors influencing spiritual needs, explore the interaction between variables by structural equation model, and provide a certain reference for developing spiritual care for people with stroke.

## Methods

### Design and setting

This cross-sectional study was performed on people with stroke from August 2022 to January 2023. This study was done in the affiliated hospitals of Hunan University of Chinese Medicine in Changsha, China.

### Participants

A cluster sampling method would be used to select people with stroke admitted for inpatient treatment in the neurology department and rehabilitation department of the affiliated hospitals of Hunan University of Chinese Medicine in Changsha, Hunan Province, China. The participants must meet this study’s inclusion and exclusion criteria, be willing to participate, and sign an informed consent form.

Participants comprised adults aged over 18 years, diagnosed with stroke by neurology specialists, and were in the recovery period or post-sequelae period. Participants understood the questionnaire and questions, gave informed consent, and voluntarily participated in this study.

Exclusion criteria included individuals with conditions that would hinder their ability to participate in the survey, such as language barriers, hearing impairments preventing cooperation with the investigator, and mental illness or dementia. Additionally, individuals with other significant diseases like malignant tumors, congestive heart failure, or end-stage kidney disease were also excluded.

The study included 23 explanatory variables (18 demographic variables, spiritual needs, anxiety, depression, family support, and quality of life.). According to Kendall’s sample size estimation method [17], the sample size is at least 5 to 10 times the number of independent variables, which should be 115 to 230 samples. Considering 20% loss to follow-up rate and sampling error, the sample size was expanded to 288. Our study eventually included 422 samples.

### Measures

Five questionnaires were utilized in the study to gather sociodemographic information and assess the spiritual needs, quality of life, anxiety and depression, and family support of stroke patients.

### General information questionnaire

Researchers designed a general information questionnaire to collect sociodemographic characteristics of patients, such as gender, age, ethnicity, education, residence status, mode of living, and number of children. Part of the data in our study referenced medical records, such as the patient’s stroke type, number of stroke attacks, and duration of the disease.

### Spiritual needs questionnaire (SPNQ)

The SPNQ questionnaire was developed by Arndt Büssing [18] in 2010 and is mainly used to assess the spiritual, socio-psychological, and existential needs of patients with chronic illnesses. The entire Chinese scale, which Zhao [19] amended in 2019, was used in this investigation. It consists of six dimensions, including Positive Giving, Belief and Blessing, Inner Peace, Belief Resources, Existential Reflection, and Existential Acceptance, with 27 items. A four-point Likert scale, with 0 representing little need and 3 representing high need, was employed for rating. Higher total scores indicate stronger spiritual needs; the score goes from 0 to 81. The scale’s Cronbach’s α coefficient was 0.81. With the original author’s permission, certain questionnaire items were appropriately modified in our study in light of the characteristics and cultural backgrounds of people with stroke. The Cronbach’s α coefficient was discovered to be 0.887 upon retesting.

### The MOS36-item short form health survey (SF-36)

Developed by a medical research group in the United States, SF-36 is a universally applicable questionnaire for measuring the quality of life [20]. For this study, Chinese scholars, including Li [21], adopted the revised version of the questionnaire. The questionnaire consists of 36 items divided into eight dimensions: physical health (physical functioning, role limitations due to physical health, general health, bodily pain) and mental health (social functioning, vitality, mental health, role limitations due to emotional problems). SF-36 has different scoring methods for dimensions, mainly using the three and five Likert levels. Every dimension has a percentage score ranging from 0 to 100; higher scores correspond to greater health. The scale’s Cronbach’s α coefficient was 0.94.

### Hospital anxiety and depression scale (HADS)

The HADS was created in 1983 by Zigmond AS and Snaith RP [22] and is used to screen hospitalized patients for signs of depression and anxiety. Its validity and reliability have been demonstrated in stroke populations [23]. There are 14 items on the scale, 7 of which measure anxiety and 7 of which measure sadness. A four-level Likert scale is used to grade each item, and scores can vary from 0 to 21. Following are the categories for the total score: ≤7 denotes normal, 8–10 mild abnormality, 11–14 moderate abnormalities, and ≥ 15 severe abnormalities.

### Family support self-rating scale (PSS-Fa)

Developed by Procidano and Heller [24] in the United States, the PSS-Fa was adopted for this study using the Chinese version by Yang [25] in 2011. The scale consists of 15 items; the total score ranges from 0 to 15. Scores ≤ 5 indicate low levels of family support, scores 6–10 indicate moderate levels of family support, and scores 11–15 indicate high levels of family support. This scale has been previously used in research studies involving patients with chronic illnesses [26], and its reliability and validity have been confirmed.

### Data collection

On-site recruitment of stroke patients was conducted in the hospital’s neurology and rehabilitation departments following the approval of our study by the Ethics Committee, the hospital’s nursing department, and the department managers. Before the formal investigation, four nursing postgraduates in our research group acted as the investigators and received standardized training. Throughout the data collection procedure, the investigators gave stroke patients an explanation of the study’s significance and goal, and they were given clear instructions on how to participate. Data was gathered via paper-based surveys. Our study was conducted during participants’ discretionary time throughout the day, and they were required to complete paper questionnaires on-site for confirmation and validation. The participants’ identities were kept anonymous. The research team leader will oversee the management of paper questionnaires, while two other team members will input and cross-verify data using Stata 25.0 software. Additionally, 20% of completed questionnaires were randomly checked for accuracy by another team member to ensure data quality.

### Data analysis

Following data collection, SPSS 25.0 software was used for data processing and analysis, and an Excel database was used to enter the data. Setting α = 0.05 as the significance level, all p-values shown are two-tailed probability. It was deemed statistically significant when *p* < 0.05. To characterize the demographic data, descriptive statistics like frequencies and percentages were utilized., including clinical data related to the disease, caregiving, self-satisfaction, and family support. For spiritual needs, quality of life, anxiety, depression, and family support scores, normality tests were performed, and non-normal distribution data were expressed using the median and interquartile range (M(Q)). When comparing the spiritual needs scores with demographic data, clinical data related to the disease, caregiving, self-satisfaction, and family support, Non-parametric tests such as the Wilcoxon rank-sum test and the Kruskal-Wallis H test were used if the spiritual needs ratings in each group did not follow a normal distribution. To investigate the factors influencing the spiritual requirements of patients with stroke, multiple linear regression analysis was used, with the total spiritual needs score serving as the dependent variable and variables that demonstrated statistical significance in univariate and correlation analysis as independent variables. The mediating role of family support, depression, and anxiety on the spiritual demands and quality of life of stroke patients was examined using AMOS 24.0.

### Ethical considerations

This study has obtained approval from the Ethics Committee of the First Affiliated Hospital of Hunan University of Chinese Medicine (Approval No. N-LL-YJSLW-2022-301). All stroke patients in our study consented and participated in this study voluntarily.

## Results

### Participant characteristics

This study comprised 422 people with stroke in total, Men accounted for 70.4% (*n* = 297) of the total, 65.4% (*n* = 276) of stroke patients were over 60 years of age, and 88.9% (*n* = 375) were married. The demographic characteristics of the stroke patients who participated in this study are detailed in Table 1 and Supplementary Table 1. There were statistically significant differences in spiritual needs scores among gender, marital status, primary caregiver, the main source of disease-related expenses, stroke type, number of disease occurrences, disease duration, the ability for activities of daily living, and satisfaction with psychological care and self-value realization after the disease.

**Table 1:** General Information and differences in spiritual needs scores among stroke patients(*N* = 422)

Characteristics	Frequency(*n*)	Percentage(%)	Score [M(Q1-Q3)]	H/Z	*p*-values
Gender					
Male	297	70.4	37(32–40)	-1.970^#^	0.049
Female	125	29.6	38(34 − 30)		
Marital status					
Unmarried	5	1.2	34(32.5–39.5)	13.722	0.0038
Married	375	88.9	37(32–40)		
Divorced or separated	6	1.4	37.5(36.25–40.25)		
Widowed	36	8.5	39(37–41)		
Primary caregiver					
Family Member	230	54.5	36.5(32–39)	13.526	0.004
Nanny or Caregiver	137	32.5	38(35–40)		
Self	46	10.9	35(29–39)		
Other	9	2.1	38(35–40)		
The main source of income					
Children/Spouse or other relatives	197	46.7	38(32–40)	11.337	0.003
Personal income	221	52.4	37(33–39)		
National or social assistance/subsidies	4	0.9	42.5(40.25-44)		
Type of stroke					
Ischemic stroke	298	70.6	37(31–39)		
Hemorrhagic stroke	110	26.1	37(33.75-40)	4.484	0.014
Mixed stroke	14	3.3	39(36-41.25)		
Number of stroke incidences					
1	348	82.5	37(32–39)		
2	56	13.3	38(35–40)	24.816	<0.001
3	15	3.5	40(39–43)		
4	3	0.7	43(40 − 0)		
Disease course					
0.5 months ≤ Disease course <1 months	71	16.8	35(31–39)		
1 months ≤ Disease course <6 months	176	41.7	36(32–39)	19.323	0.001
6 months ≤ Disease course <1 year	48	11.4	36(34.25-40)		
1 year ≤ Disease course < 3 years	50	11.9	38(36–41)		
Disease course ≥ 3 years	77	18.2	39(34.5–40)		
The activity of daily living					
No dependence	83	19.7	31(28–36)	82.496	<0.001
Mild dependence	125	29.6	36(32.25-39)		
Moderate dependence	129	30.6	38(34.75-41)		
Heavy dependence	85	20.1	39(36.5–41)		
Psychological care					
Very satisfied	14	14	32.5(27.75–40.75)	59.222	<0.001
Satisfied	172	172	35(31–38)		
Average	176	176	38(34.25-40)		
Dissatisfied	53	53	40(36.25-41)		
Very dissatisfied	7	7	42(41–44)		
Realization of self-worth after stroke					
Very satisfied	3	3	32(27 − 0)	93.783	<0.001
Satisfied	17	17	32(26.75–34.5)		
Average	153	153	34(30–38)		
Dissatisfied	193	193	38(35–41)		
Very dissatisfied	56	56	40(37.25–41.75)		

Note: # represents the z-value, and the rest are H-values. M represents the median, and Q represents the quartiles

### Descriptive statistics of spiritual needs, anxiety and depression, quality of life, and family support by people with stroke

The mean and standard deviation of the total score and the dimension score for each study variable are shown in Table 2 and Supplementary Table 2. The results of this study showed that the spiritual needs scores of the included people with stroke ranged from 20 to 47, with a median score of 37 [IQR 33 to 40)], indicating a moderate level, and the dimension with the highest score was positive, with a median score of 12 [IQR 11 to 13)].

**Table 2:** Scores of spiritual needs in people with stroke (*N* = 422)

Items	Range	Score [M(Q1-Q3)]
Existential reflection	1–12	8(7–9)
Inner peace	3–13	8(7–9)
Positive giving	5–16	12(11–13)
Belief and prayer	0–9	6(4–7)
Acceptance	0–4	2(0–2)
Resources of faith	0–7	1(0–3)
Total score of spiritual needs	20–47	37(33–40)
Total quality of life score	19.56-94	39.75(33.35–52.41)
Total family support score	5–15	13(12–15)

### Correlation analysis of people with stroke’s spiritual needs, quality of life, anxiety and depression, and family support

Spearman correlation analysis revealed that the spiritual needs of stroke patients were significantly negatively correlated with quality of life (*r*=-0.469, *p* < 0.01), significantly negatively correlated with family support (*r*=-0.163, *p* < 0.01), and significantly positively correlated with anxiety (*r* = 0.479, *p* < 0.01) and depression (*r* = 0.493, *p* < 0.01). Detailed results can be found in Table 3.

**Table 3:** Correlation analysis of spiritual needs, quality of life, anxiety, depression, and family support in people with stroke (R-values)

	Spiritual needs	Existential reflection	Inner peace	Positive giving	Belief and prayer	Acceptance	Resources of faith
Quality of life	-0.469^**^	-0.458^**^	-0.053	-0.188^**^	-0.401^**^	0.025	-0.213^**^
Anxiety score	0.479^**^	0.579^**^	0.111^*^	0.187^**^	0.337^**^	-0.214^**^	0.154^**^
Depression score	0.493^**^	0.569^**^	0.121^*^	0.205^**^	0.346^**^	-0.128^**^	0.191^**^
Family support	-0.163^**^	-0.270^**^	-0.010	-0.091	-0.065	0.282^**^	-0.115^*^

Note: ** indicates a significant correlation at the 0.01 level (two-tailed), and * indicates a significant correlation at the 0.05 level

### Multiple linear regression analysis of influencing factors of spiritual needs in people with stroke

The results of multiple linear regression analysis showed that primary economic sources for disease-related expenditures (*p* = 0.044), number of stroke occurrences (*p* = 0.001), duration of illness (*p* = 0.023), activities of daily living (*p* = 0.006), depression(*p* = 0.034), and family support(*p* = 0.008) were all factors that affected the spiritual needs score. Please refer to Table 4 for details.

**Table 4:** Multivariate analysis of spiritual needs in people with stroke (*N* = 422)

Variable	β	SE	β´	t	*p*-values
Constant	13.756	4.307	—	3.194	0.002
Primary economic sources for disease-related expenditures					
Children/spouse or other relatives	0				
Personal income	-0.102	0.495	-0.01	-0.207	0.836
National or social assistance/subsidies	4.382	2.173	0.083	2.016	0.044
Number of stroke occurrences	1.529	0.464	0.159	3.295	0.001
Duration of Illness	0.426	0.187	0.113	2.28	0.023
The activity of daily living	0.903	0.329	0.178	2.748	0.006
Depressive score	0.299	0.14	0.16	2.126	0.034
Family support score	0.39	0.146	0.146	2.672	0.008

Note: R^2^ = 0.414; Adjusted R^2^ = 0.364, F = 8.293, *P* = 0.000

### Mediating roles between spiritual needs and quality of life, anxiety, and depression, and family support in stroke patients

**Fig. 1:**
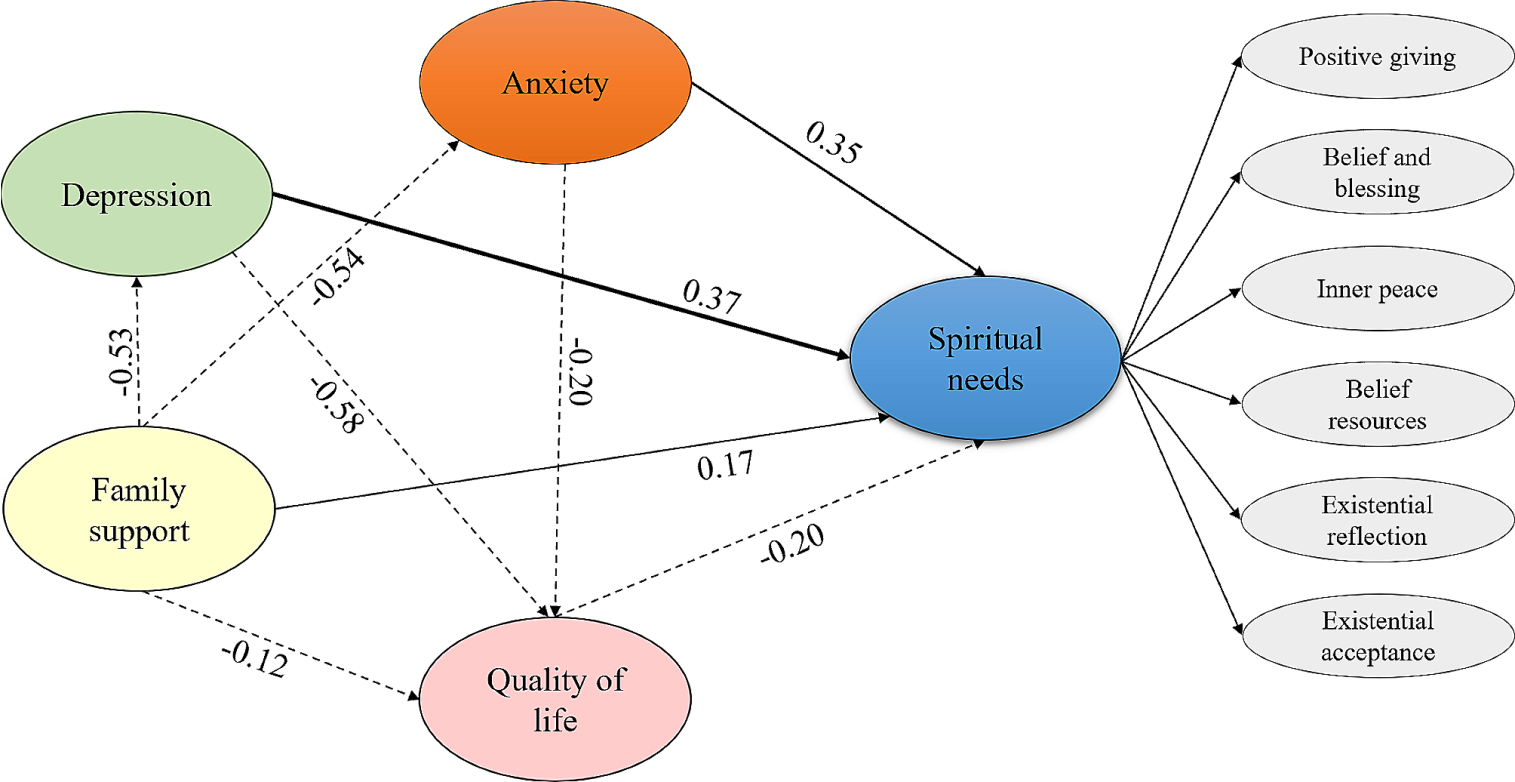
Mediation model of spiritual needs, quality of life, anxiety and depression, and family support in people with stroke. Note: The dotted line represents the negative effect, the solid line represents the positive effect, and the bold solid line represents the strongest positive effect

The effect relationship amongst factors in the fitting model is shown in Fig. 1, and the detailed path analysis results are shown in Table 5. The mediation effects revealed that depression (β = 0.368, *p* = 0.005) had the most potent positive effect on spiritual needs, followed by anxiety (β = 0.347, *p* = 0.004), and family support (β = 0.167, *p* = 0.023). Quality of life (β=-0.202, *p* = 0.017) had negative effects on spiritual needs. Both anxiety, depression, and family support can directly or indirectly influence spiritual needs, while quality of life has a direct effect, the details are shown in Supplementary Table 3.

**Table 5:** Path analysis results of spiritual needs, quality of life, anxiety, depression, and family support in people with stroke

Path	Standardized Path Coefficient	S.E.	C.*R*.(t values)	*p*-values
Family support → Depression	-0.530	0.059	-12.838	<0.001
Family support → Anxiety	-0.544	0.067	-13.293	<0.001
Depression → Quality of life	-0.583	0.278	-10.259	<0.001
Family support → Quality of life	-0.116	0.301	-2.697	0.007
Anxiety → Quality of life	-0.201	0.246	-3.5	<0.001
Quality of life → Spiritual needs	-0.202	0.002	-2.383	0.017
Anxiety → Spiritual need	0.347	0.014	2.867	0.004
Depression → Spiritual needs	0.368	0.017	2.836	0.005
Family support → Spiritual needs	0.167	0.013	2.278	0.023

## Discussion

In this study, the median total score of spiritual needs among people with stroke was 37, indicating a moderate level of spiritual needs. This result is similar to the findings of Büssing [27], which may be related to the type of disease as both fall within the category of chronic illnesses. However, there are differences between the results of this study and the studies by Frick [28] and Zhao [19]. Frick’s study focused on patients in the emergency department who had varying degrees of disease severity and shorter duration of illness, resulting in lower levels of spiritual needs. On the other hand, Zhao Yue’s study concentrated on cancer patients, who experienced higher degrees of psychological discomfort and suffering due to more significant threat to their lives than people with stroke. In the spiritual dimension of this study, the highest score is given to the positive giving dimension, which is higher than the study by VALENTE [29]. This may be attributed to the cultural characteristics of Chinese people with stroke. Traditional Chinese culture advocates Confucianism, emphasizing “benevolence, righteousness, and dedication.” In this cultural atmosphere and spiritual influence, the demand for positive giving is expected to be higher. Furthermore, the mental challenges of recovery and potential relapse are often experienced by stroke survivors, who seek to reestablish connections with their family and community through self-guided efforts, achieve a sense of self-worth, and prevent themselves from becoming a burden.

In this study, the frequency of stroke, duration of illness, activities of daily living, and family support were identified as significant factors influencing the spiritual needs of people with stroke. The results show that the higher the frequency of stroke occurrence, the higher the spiritual needs of the patients. Previous studies have indicated that patients who experience recurrent strokes are better able to cope with the disease because they have accumulated knowledge and experience from their initial stroke episode [30]. As a result, they are more prone to turn to outside assistance, which raises their spiritual demands. On the other hand, the duration of illness hurts the spiritual needs of patients, which further validates previous research findings [14]. Stroke recovery is a long-term process, and studies have shown that a longer duration of illness hurts social participation, depressive symptoms, and neurological function of people with stroke [31]. The longer the duration of the illness, the higher the level of inner suffering and the stronger the desire to seek solutions, resulting in higher spiritual needs. In this search, the degree of family support was found to have a significant impact on the spiritual needs of people with stroke. Family factors play a significant role in determining a patient’s capacity for self-management, and better health behaviors are associated with higher levels of family support. [32]. Therefore, people with stroke with higher levels of family support experience higher levels of happiness, have a more positive attitude towards coping with the disease and are more able to accept help and find solace from family members, resulting in lower levels of spiritual distress and lower spiritual needs. Patients with higher levels of impairment had higher spiritual needs, according to this study, which assessed patients’ degrees of disability based on how well they performed in daily activities. Considering that stroke patients with disability often have low levels of self-care ability and most of them rely heavily on assistance in daily life, they may develop self-doubt and have lower self-worth [33]. Thus, they hope to explore pathways to realize their value better.

Although previous studies have supported the impact of family support and negative emotions on the spiritual needs of patients [34, 35], the underlying mechanisms remain unclear. Thus, this study examines the relationship between the level of spiritual needs, anxiety, depression, and family support in people with stroke through the analysis of mediating effects. Stroke is an abrupt, bad life event that has serious aftereffects and leaves high percentage of disabled people. Patients often experience worry and sadness as a result, which might change how they manage their illness [1]. The results of this study indicate the more severe the anxiety and depression, the higher the spiritual needs. Patients with higher levels of anxiety frequently turn to other people for conversation as well as various forms of spiritual and emotional support, which can aid in rediscovering life’s purpose and meeting other spiritual needs. Therefore, anxiety and depression can influence the level of spiritual health and further affect the spiritual needs of patients [36]. The results show that family support can directly affect spiritual needs, indicating that the lower the levels of family support, the higher the spiritual needs. Family members’ empathy, compassion, and tolerance can offer patients rich emotional engagement and substantial spiritual support, assisting them in re-establishing their faith in their ability to overcome illnesses, accepting who they are in the moment, and easing their transition into the outside world, thus expanding their perspective on the purpose of life [14], which is consistent with previous research [37]. Furthermore, apart from its direct effect, family support can also indirectly affect spiritual needs through anxiety, depression, and quality of life. Studies have shown that good social support and family care act as protective factors against anxiety and depression [38]. Social support can regulate individuals’ psychological distress, thereby reducing the adverse effects of stress, relieving negative emotions, and improving patients’ spiritual health [39]. Therefore, when addressing patients’ spiritual issues, healthcare professionals should consider their spiritual condition and the influence of their emotional state.

It is important to note that we also discovered variations in the spiritual requirements of people with stroke based on various demographic factors. This study found significant differences (*p* < 0.05) between genders in the spiritual needs scores. The spiritual needs score for women is higher than that of men. Women are typically more emotionally sensitive than men due to physiological variations. When experiencing negative emotions, female patients are more inclined to express their feelings and seek help and solace from those around them [40]. This result is in line with earlier studies [41]. Likewise, this study implies that patients who have experienced a loss may have more significant spiritual needs. Spouses are a vital part of an individual’s life, and a high-quality marital status positively impacts physical and mental health [42]. Patients with various caregivers score differently on spiritual requirements, which deviates from earlier research [19]. Previous research mainly focused on family members as caregivers, whereas this study involved different intimate relationship situations. When the caregiver is a family member, the relationship between the patient and caregiver is closer, leading to more robust spiritual needs. However, When the caregiver is a full-time carer employed, the relationship is more distant, and patients do not often express their inner thoughts to them. Therefore, patients under the care of nurses or caretakers have higher spiritual needs. Moreover, this study showed that patients with mixed strokes had higher spiritual needs. Mixed stroke, with a combination of hemorrhagic stroke and ischemic stroke, has higher risk factors and a more significant impact from the disease, resulting in a relatively poorer prognosis. Hence, their spiritual needs are more vital, consistent with previous research [43].

It is necessary to pay attention to the spiritual needs of stroke patients, the hospital can establish a specialized team to assess spiritual needs and provide tailored spiritual care to assist patients feel more confident and capable of actively managing their illness. nurse should consider their patients’ emotional state and spiritual condition when managing their spiritual concerns. To boost patients’ confidence in beating the illness, nurses should give them compassionate medical attention, pay attention to their negative feelings like worry and sadness, and provide prompt emotional support and support in fulfilling their spiritual requirements. In our study, we demonstrated that the spiritual needs of people in China with stroke were at an intermediate level, influenced by factors such as stroke frequency, duration, and negative emotion. Anxiety, depression, and level of family support can directly or indirectly affect a patient’s spiritual needs, and quality of life had a direct effect on spiritual needs, by identifying the needs of patients and providing them with effective and comprehensive spiritual care, to promote the development and progress of spiritual care in China.

### Limitations

This study has limitations: (1) Because this is a cross-sectional study, more excellent care should be taken in interpreting the causal relationship between the variables in the findings. more precise control of variables will be used to eliminate the influence of confounding variables (such as degrees of disability) as much as possible in the future and relevant longitudinal studies can be conducted to explore the trajectory of spiritual needs and the causal relationships between more variables. (2) The limited inclusion of people with stroke from the affiliated hospitals of Hunan University of Chinese Medicine in this study may introduce selection bias and restrict its applicability, Subsequent investigators contemplate broadening the sample’s geographic range to establish a multi-center and multi-regional investigation. (3) Results may be skewed since the patients may not have had the same understanding of “spirituality” due to their varied cultural origins. It is recommended that spirituality and associated concepts be more standardized within a set cultural framework. And the choices of individuals with no belief in spirituality should also be valued.

## Conclusion

The results of this study showed that people with stroke have a moderate level of spiritual needs in China, which is also affected by many factors. It is recommended that clinical nurses, family members, and society recognize people with stroke’s spiritual needs and provide targeted spiritual care to improve patients’ support level, psychological health, quality of life, and satisfaction and facilitate disease recovery. This study was a descriptive cross-sectional one, we need more work on how to improve spiritual care for stroke patients in the future, further intervention research can be conducted in different cultural backgrounds to construct appropriate spiritual care plans.

### Electronic supplementary material

Below is the link to the electronic supplementary material.

Supplementary Material 1

## Data Availability

The datasets used and/or analysed during the current study available from the corresponding author on reasonable request.
