# Supplementary material for: Spiritual needs and influencing factors among people with stroke in China: a cross-sectional study
Source: BMC Nurs. 2024 Jul 29;23:507. doi: 10.1186/s12912-024-02182-7 (PMC11287944; doi:10.1186/s12912-024-02182-7)
Supplement: Supplementary file 1 — Supplementary Material 1 [file 12912_2024_2182_MOESM1_ESM.docx]

**Supplementary Table 1 General Information and differences in spiritual needs scores among stroke patients（supplement）（N=422）**

| **Characteristics** | **Frequency（n）** | **Percentage（%）** | **Score**  **[M(Q1-Q3）]** | **H/Z** | **p-values** |
| --- | --- | --- | --- | --- | --- |
| Gender |  |  |  |  |  |
| Male | 297 | 70.4 | 37(32-40) | -1.970^#^ | 0.049 |
| Female | 125 | 29.6 | 38(34-30) |  |  |
| Nationality |  |  |  |  |  |
| Han | 413 | 97.9 | 37(33-40) | -0.253^#^ | 0.800 |
| Minority | 9 | 2.1 | 37(34-40) |  |  |
| Age（years） |  |  |  |  |  |
| ≤40 | 15 | 3.6 | 35(32-39) | 0.528 | 0.768 |
| 41-59 | 131 | 31.0 | 37(34-39) |  |  |
| ≥60 | 276 | 65.4 | 37(31.25-40) |  |  |
| Marital status |  |  |  |  |  |
| Unmarried | 5 | 1.2 | 34(32.5-39.5) | 13.722 | 0.0038 |
| Married | 375 | 88.9 | 37(32-40) |  |  |
| Divorced or separated | 6 | 1.4 | 37.5(36.25-40.25) |  |  |
| Widowed | 36 | 8.5 | 39(37-41) |  |  |
| Education level |  |  |  |  |  |
| Primary school and below | 57 | 13.5 | 37(33-40) | 4.655 | 0.199 |
| Junior school | 151 | 35.8 | 38(33-40) |  |  |
| High school or Junior college | 120 | 28.4 | 37(31.25-39) |  |  |
| College and above | 94 | 22.3 | 36(32.75-39) |  |  |
| Residence place |  |  |  |  |  |
| Rural | 65 | 15.4 | 37(34-40) | 2.744 | 0.250 |
| Urban | 101 | 23.9 | 36(32-39) |  |  |
| City | 256 | 60.7 | 37(32-40) |  |  |
| Residence status |  |  |  |  |  |
| Alone | 26 | 6.2 | 38(34.75-41.25) | 5.289 | 0.071 |
| With Family | 394 | 93.4 | 37(32-40) |  |  |
| Welfare Institution or Collective | 2 | 0.5 | 41(40-0) |  |  |
| Primary caregiver |  |  |  |  |  |
| Family Member | 230 | 54.5 | 36.5(32-39) | 13.526 | 0.004 |
| Nanny or Caregiver | 137 | 32.5 | 38(35-40) |  |  |
| Self | 46 | 10.9 | 35(29-39) |  |  |
| Other | 9 | 2.1 | 38(35-40) |  |  |
| Number of children |  |  |  |  |  |
| 0 | 13 | 3.1 | 37(34.5-41.5) | 4.552 | 0.210 |
| 1 | 183 | 43.4 | 37(32-39) |  |  |
| 2 | 164 | 38.9 | 37(33.25-40) |  |  |
| ≥3 | 62 | 14.7 | 38(32.75-41) |  |  |
| Monthly income per capita (RMB) |  |  |  |  |  |
| ＜2000 | 19 | 4.5 | 39(37-41) | 6.953 | 0.073 |
| 2000-3999 | 128 | 30.3 | 37(33.25-40) |  |  |
| 4000-5999 | 201 | 47.6 | 37(32-40) |  |  |
| ≥6000 | 74 | 17.5 | 37(32.75-39) |  |  |
| Other | 2 | 0.5 | 35.5(25-0) |  |  |
| The main source of income |  |  |  |  |  |
| Children/Spouse or other relatives | 197 | 46.7 | 38(32-40) | 11.337 | 0.003 |
| Personal income | 221 | 52.4 | 37(33-39) |  |  |
| National or social assistance/subsidies | 4 | 0.9 | 42.5(40.25-44) |  |  |
| Type of stroke |  |  |  |  |  |
| Ischemic stroke | 298 | 70.6 | 37（31-39） |  |  |
| Hemorrhagic stroke | 110 | 26.1 | 37(33.75-40) | 4.484 | 0.014 |
| Mixed stroke | 14 | 3.3 | 39(36-41.25) |  |  |
| Number of stroke incidences |  |  |  |  |  |
| 1 | 348 | 82.5 | 37(32-39) |  |  |
| 2 | 56 | 13.3 | 38(35-40) | 24.816 | ＜0.001 |
| 3 | 15 | 3.5 | 40(39-43) |  |  |
| 4 | 3 | 0.7 | 43(40-0) |  |  |
| Disease course |  |  |  |  |  |
| 0.5 months ≤ Disease course ＜1 months | 71 | 16.8 | 35（31-39） |  |  |
| 1 months ≤Disease course ＜6 months | 176 | 41.7 | 36(32-39) | 19.323 | 0.001 |
| 6 months ≤ Disease course ＜1 year | 48 | 11.4 | 36(34.25-40) |  |  |
| 1 year ≤ Disease course ＜ 3 years | 50 | 11.9 | 38(36-41) |  |  |
| Disease course ≥3 years | 77 | 18.2 | 39(34.5-40) |  |  |
| Activities of daily living |  |  |  |  |  |
| Able to perform self-care | 83 | 19.7 | 31(28-36) | 82.496 | ＜0.001 |
| Mild dependency | 125 | 29.6 | 36(32.25-39) |  |  |
| Moderate dependency | 129 | 30.6 | 38(34.75-41) |  |  |
| Unable to perform self-care | 85 | 20.1 | 39(36.5-41) |  |  |
| Daily life care |  |  |  |  |  |
| Very satisfied | 19 | 4.5 | 33(31-40) | 7.101 | 0.069 |
| Satisfied | 342 | 81 | 37(33-39) |  |  |
| Average | 54 | 12.8 | 38(35-41) |  |  |
| Dissatisfied | 7 | 7 | 38(26-41) |  |  |
| Very dissatisfied | 0 | 0 |  |  |  |
| Psychological care |  |  |  |  |  |
| Very satisfied | 14 | 14 | 32.5(27.75-40.75) | 59.222 | ＜0.001 |
| Satisfied | 172 | 172 | 35(31-38) |  |  |
| Average | 176 | 176 | 38(34.25-40) |  |  |
| Dissatisfied | 53 | 53 | 40(36.25-41) |  |  |
| Very dissatisfied | 7 | 7 | 42(41-44) |  |  |
| Realization of self-worth after stroke |  |  |  |  |  |
| Very satisfied | 3 | 3 | 32(27-0) | 93.783 | ＜0.001 |
| Satisfied | 17 | 17 | 32(26.75-34.5) |  |  |
| Average | 153 | 153 | 34(30-38) |  |  |
| Dissatisfied | 193 | 193 | 38(35-41) |  |  |
| Very dissatisfied | 56 | 56 | 40(37.25-41.75) |  |  |

Note: # represents the z-value, and the rest are H-values. M represents the median, and Q represents the quartiles.

**Supplementary Table 2 Spiritual needs of stroke patients with different levels of anxiety and depression (N=422)**

| **Items** | **Number（%）** | **Spiritual needs score**  **[M(Q1-Q3)]** | **H** | **p-values** |
| --- | --- | --- | --- | --- |
| Anxiety levels |  |  |  |  |
| Normal | 257(60.9%) | 35(31-38) | 72.808 | ＜0.001 |
| Mild | 97(23%) | 38(35-41) |  |  |
| Moderate | 66(15.6%) | 40(39-41) |  |  |
| Severe | 2(0.5%) | 43(43-43) |  |  |
| Depression levels |  |  |  |  |
| Normal | 200(47.4%) | 34(30-38) | 91.570 | ＜0.001 |
| Mild | 143(33.9%) | 38(35-40) |  |  |
| Moderate | 74(17.5%) | 40(38-41) |  |  |
| Severe | 5(1.2%) | 41(38.5-43) |  |  |

**Supplementary Table 3 Significance test results of path coefficients for each variable**

| **Dependent variable** | **Effects** | **Independent variables** | | | |
| --- | --- | --- | --- | --- | --- |
|  |  | **Anxiety** | **Depression** | **Family support** | **Quality of life** |
| Spiritual needs | Direct effect | 0.347 | 0.368 | 0.167 | -0.202 |
|  | Indirect effects | 0.041 | 0.118 | -0.445 | 0.000 |
|  | Total effect | 0.388 | 0.486 | -0.278 | -0.202 |
